# Cancer therapeutics using survivin BIRC5 as a target: what can we do after over two decades of study?

**DOI:** 10.1186/s13046-019-1362-1

**Published:** 2019-08-22

**Authors:** Fengzhi Li, Ieman Aljahdali, Xiang Ling

**Affiliations:** 1 Department of Pharmacology & Therapeutics, Roswell Park Comprehensive Cancer Center, Elm and Carlton Streets, Buffalo, New York 14263 USA; 2 Department of Cellular & Molecular Biology, Roswell Park Comprehensive Cancer Center, Elm and Carlton Streets, Buffalo, New York 14263 USA; 3 Developmental Therapeutics Program, Roswell Park Comprehensive Cancer Center, Elm and Carlton Streets, Buffalo, New York 14263 USA; 4 Canget BioTekpharma LLC, Buffalo, New York USA

**Keywords:** Survivin cancer therapeutics, survivin, BIRC5, Mcl-1, XIAP, cIAP2, MdmX, YM155, FL118, survivin immunotherapy

## Abstract

Survivin (also named BIRC5) is a well-known cancer therapeutic target. Since its discovery more than two decades ago, the use of survivin as a target for cancer therapeutics has remained a central goal of survivin studies in the cancer field. Many studies have provided intriguing insight into survivin’s functional role in cancers, thus providing promise for survivin as a cancer therapeutic target. Despite this, moving survivin-targeting agents into and through the clinic remains a challenge. In order to address this challenge, we may need to rethink current strategies in order to develop a new mindset for targeting survivin. In this Review, we will first summarize the current survivin mechanistic studies, and then review the status of survivin cancer therapeutics, which is classified into five categories: (i) survivin-partner protein interaction inhibitors, (ii) survivin homodimerization inhibitors, (iii) survivin gene transcription inhibitors, (iv) survivin mRNA inhibitors and (v) survivin immunotherapy. We will then provide our opinions on cancer therapeutics using survivin as a target, with the goal of stimulating discussion that might facilitate translational research for discovering improved strategies and/or more effective anticancer agents that target survivin for cancer therapy.

## Background

Drs. Wheatley and Altieri recently summarized the major accomplishments achieved with survivin studies that have taken place over the past 21 years [1]. This authoritative summary of survivin studies will be useful for directing future studies on the basic biology and molecular mechanism of action of survivin, and thus will allow the survivin research community to renew and reconsider our approaches to certain sub-areas of survivin studies in the coming decade.

Drs. Wheatley and Altieri’s final conclusion in their review article expressed that in the wake of the 21st anniversary of its discovery, our knowledge of survivin has expanded exponentially, yet we still do not have a survivin-specific anticancer agent [1]. The most advanced survivin cancer therapeutic agents, survivin antisense oligonucleotides and YM155, were halted after multiple clinical trials due to either low antitumor efficacy and/or over toxicity issues. In this review, we will focus on this key challenge area. We will review and update the publications relevant to survivin, as well as provide our opinions based on existing observations in literature that are relevant to targeting survivin for drug discovery and molecular cancer therapeutics.

## Current status of survivin studies

Survivin lies at the crossroads of a number of cancer cell signaling networks. Specifically, many upstream cellular signaling molecules control and regulate survivin and its functions. These upstream signaling molecules constitute survivin’s incoming networks. The upstream molecules include: binding protein, protein regulator, various enzymes (protease, kinase, phosphatase), transcription factor, miRNA, transporter and channel protein, receptor with or without kinase activity, and their corresponding ligands (Table 1). Survivin is also able to control and regulate many of the upstream molecules listed above and/or other molecules to form its outgoing network (Table 2). While the findings in Tables 1 and 2 cover a broad area of past and current investigations into survivin’s mechanisms of action, further study is needed in many of the identified subareas in order for the findings to be of practical use for molecular cancer therapeutics. For example, in the survivin incoming network (Table 1), many of these findings need further investigation to confirm the significance of their interaction and consequences in cancer before being used for cancer therapeutic strategy design. A similar situation exists for the survivin outgoing network, as shown in Table 2. In this regard, while a great deal of knowledge about survivin has been accumulated over the past two decades [1], there is still much more work that needs to be done on survivin mechanistic studies in many aspects of the subareas listed in Tables 1 and 2. Further in-depth studies would strengthen the foundation for designing better survivin-relevant cancer therapeutics.

**Table 1:** Survivin incoming signal network^a^: molecules that regulate survivin (drugs’ effects on survivin not included)

Mechanism and Effects	Classified Factors/Molecules
1. Binding to survivin for activation	**Binding protein:** Beclin 1, CDCA8, CUGBP1, DBC1, ELAVL1 (HuR), FAT10, HBXIP, HSP60, HSP90, MKLP2, NET1 (TSPAN1), VIL2 (ezrin). **Protein:** C6orf176; **Enzyme:** cIF2C2 (Argonaute-2), PARP6, RecQL4, SFRS1 (SF2). **Regulator:** Evi5. **Protease:** UFD1.
2. Phosphorylation of survivin for activation	**Kinase:** Aurora-B, Casein Kinase II alpha chains, CDK1 (p34), PLK1.
3. Deubiquitination of survivin for activation	**Enzyme:** JAB1.
4. Transportation of survivin for activation	**Transporter:** CRM1.
5. Transcriptional upregulation of survivin for activation	**Transcription factor:** AR, b-Myb, c-Myb, c-Myc, CREB1, DEC1 (stra13), E2F1, E2F3, EPAS1, ESR1, FBI-1(Pokemon), FOXM1, GATA-1, HIF-1, HIF1A, JunB, KLF5, Lef-1, N-Myc, NF-κB, NF-κB p50/p65, NUR77, PPAR-γ, RBP-Jκ (CBF1), NF-κB Rel A/p65, NF-κB Rel B, RUNX2, SOX2, SOX4, SP1, SP3, STAT1, STAT3, TBX5, Tcf (Lef), TCF7L2 (TCF4), TEF-1, TEF-4, TFAM, THAP1, ZFX, EBNA1 (HHV4), NOTCH1 (NICD). **Binding protein:** β-arrestin1, β-catenin, LIN-9, MBD3, YAP1 (Yap65,). **Enzyme:** CBP, HDAC3, LSD1, WHSC1. **Protein:** LANA (HHV8).
6. Influence on survivin expression for activation	**Kinase:** AKT2, JNK (MAPK8-10). **Receptor ligand:** Angiopoietin1, Angiopoietin 2, Angiotensin II, Apo-2L (TNFSF10), BMP6, BMP7, CCL2, Choriogonadotropin, CTGF, EGF, Epo, FGF10, FGF19, FGF2, FLT3 ligand, FSH, G-CSF, Gastrin 17-Gly, GM-CSF, HDGF, IFN-γ, IGF-1, IL-1α, IL-2, IL-22, IL-3, IL-33, IL-5, Jagged1, Leptin, MGF, TGF-β, Thrombopoietin, TNF-α, VEGF-A, WNT3A. **Transcription factor:** ARNT, FOXK1, HoxC10, ID1, NANOG, SIP1 (ZFHX1B), SNAIL1, SP4, STAT5A, STAT5B, TCF8. **Receptor:** CD44, RAGE. **Binding protein:** COMP, CRP, Galetin-3, Mucin 16. **Receptor kinase:** ErbB2, TIE2. **Receptor:** FN14 (TNFRSF12A), FZD7, IL6RA, LMP1 (HHV4), TLR4. **Protein:** HCV NS5A, TCR α/β.
7. Binding to survivin for inhibition	**Binding protein:** PARC, Smac (Diablo), Ubiqutin. **Enzyme:** Pin1. **Protein:** Rhbdf2.
8. Ubiquitination of survivin for inhibition	**Binding protein:** FBXL7, XIAP.
9. Cleavage of survivin for inhibition	**Protease:** Granzyme M.
10. Transcriptional regulation of survivin for inhibition	**Transcription factor:** NF-κB c-Rel, CDX2, E2F4, EGR1, FKHR, FOXO3A, GCR, HNF1-α, HNF4-α, HOXA4, KLF4, p53, p63, PDEF, RUNX3, YY1. **Binding protein:** BMI-1, p130, p21, PML. **Enzyme:** DNMT1, G9a, HDAC6, Sirtuin1.
11. Effect-unspecified inhibition	**Enzyme:** SSAT
12. Influence on survivin expression for inhibition	**Receptor ligand:** Adiponectin, BMP4, DLL4, IL-1β, IL-24. **Transcription factor:** C/EBPδ, ESR2, IRF1, NRSF, SOX7, VDR. **Receptor:** CD30 (TNFRSF8), Dopamine D2 receptor, TLR3, TLR9. **Receptor kinase:** DR5 (TNFRSF10B,). **Phosphatase:** FHIT. **Binding protein:** RPS29, SUMO-1.
13. Competition with survivin for inhibition	**Receptor kinase:** RET
14. miRNA binding to survivin transcript for inhibition	**miRNA:** miR-130-3p, mir-138-5p, miR-143-3p, miR-145-5p, miR-146a-5p, miR-150-5p, miR-16-5p, miR195-5p, miR-203-3p, miR-214-3p, miR-218-5p, miR-222-3p, miR-320-3p, miR-320c, miR-335-5p, miR-338-5p, miR-34a-5p, miR-3613-3p, miR-483-3p, miR-485-3p, miR-494-3p, miR-497-5p, miR-542-3p, miR-542-5p, miR-548d-3p, miR-552-3p, miR-634, miR-708-5p, miR-96-5p.
15. Homodimerization for survivin stabilization	**Binding protein:** survivin
16. Binding to survivin with unspecified effects	**Binding protein:** AIP, APG5, cIAP1, cIAP2, FMIP, GSPT1, GSPT2, INCENP, TPX2, TRAP-1. **Kinase:** Aurora-C, GSK3β. **Enzyme:** Dicer. **Protein:** eIF3, GAS2L3, TD-60. **Receptor:** HLP-DRB1. **Channel:** Kir3.2. **Transporter:** SLC5A8. **Regulator:** p120GAP. **Phosphatase:** PP1-catγ. **Protein:** RED. **Transcription factor:** SFRS10, USF2, HBV-C X protein.
17. Phosphorylation of survivin with unspecified effects	**Kinase:** CDK5, PRPK.
18. Deubiquitination of survivin with unspecified effects	**Protease:** USP9X.
19. Transcription regulation of survivin with unspecified effects	**Transcription factor:** AHR, AML1 (RUNX1), AP-2, AP-2α, ATF-2, E2F2, E2F5, E2F6, E2F7, E2F8, ETS1, GABP-α, HNF1-β, LMO2, MAD, Max, MTF-1, Mxi1, MYOG, NF-Y, NFAT-90, Oct-2, RARα, RARβ, STAT6, TAL1, TCF7/TCF1, TCF7L1/TCF3, ZNF143, ZNF42/MZF1, hASH1, HCF1, NRB54. **Binding protein:** CXXC4, ING1, ING2, Rb protein, Sin3α. **Enzyme:** DPY30, HDAC1, HDAC4, JMJD2A, JMJD3, p300, PLU-1, PRMT1, RBB2.
20. Influence on survivin expression with unspecified effects	**Receptor ligand:** TGF-beta1.
21. miRNA binding to survivin transcript with unspecified effects	**miRNA:** miR-1225-3p, miR-1226-3p, miR-125s-3p, miR-135a-3p, miR-17-5p, miR-182-5p, miR-192-5p, miR-199a-5p, miR-19b-3p, miR-200a-3p, miR-206-3p, miR-27a-3p, miR-31-5p, miR-3127-5p, miR-320-5p, miR-338-3p, miR-449a-5p, miR-512-5p, miR-574-3p, miR-600, miR-669d-3p, miR-762, miR-764, miR-92a-3p, miR-let-7a-5p, miR-let-7f-5p

^a^Information was retrieved from the GeneGo database at https://portal.genego.com/ and organized by the authors. Relevant references for individual findings can be found in the database from the corresponding molecule under the “link info”.

**Table 2:** Survivin outgoing signal network^a^: molecules that are regulated by survivin

Mechanism and Effects	Classified Factors/Molecules
1. Survivin binds to the molecule for activation	**Transporter:** ABCG2. **Kinase:** Aurora-B, CDK1 (p34), CDK4, DNA-PK. **Phosphatase:** CDC25B. **Binding protein:** INCENP, NET1 (TSPAN1), XIAP.
2. Survivin transcriptionally regulates the molecule for activation	**Receptor kinase:** c-kit. **Kinase:** IKK-beta.
3. Survivin affects the molecule expression for activation	**Binding protein:** Cyclin D1, Cyclin E. **Transcription factor:** HIF1A. **Protease:** MMP-9. **Receptor ligand:** VEGF-A.
4. Survivin binds to the molecule for inhibition	**Protease:** Caspase-3, Caspase-7, Caspase-9. **Transcription factor:** STAT3.
5. Survivin transcriptionally regulates the molecule for inhibition	**Enzyme:** Dicer.
6. Unspecified inhibition	**Protease:** Caspase-8.
7. Survivin affects the molecule expression for inhibition	**Receptor-kinase:** DR5 (TNFRSF10B).
8. Homodimerization for survivin stabilization	**Binding protein:** survivin
9. Survivin binds to the molecule with unspecified effects	**Binding protein:** AIP, Bcl-2, Birc6, C7orf59k, CARD5, CDCA8, Clathrin heavy chain, Cyclin B1, Eps15, Histone H2AX, Histone H3, HSP90α, Ku70, MED18, MED29, MED6, MED8, NFBD1, p53BP1, Rb protein, Smac (Diablo), SRB7, SURF5, TRAP170, TRAP18, TRAP25, TRAP80, TRFP, TRG20, Tubulin, Tubulin-β, VDRIP. **Kinase:** Aurora-A, CDK2. **Protein:** CCDC102A. **Transcription factor:** E2F4, E2F5, TRIP2. **GTPase:** G-protein α-i3, Ran. **Transporter:** HBZ. **Protease:** HtrA2, p17 CASP3. **Protein:** JTB. **Receptor:** SSC5D.
10. Survivin transcriptionally regulates the molecule with unspecified effects	**Transcription factor:** BLIMP1 (PRDI-BF1), p53. **miRNA:** miR-520b. **Binding protein:** p21.

^a^Information was retrieved from the GeneGo database at https://portal.genego.com/ and organized by the authors. Relevant references for each finding can be found in the database from the corresponding molecule under the “link info”.

## Cancer therapeutics using survivin as a target

We previously reviewed survivin-selective inhibitors and also summarized the generalized survivin inhibitors that were found to inhibit survivin expression during their mechanistic studies [2]. An example of such generalized survivin inhibitors is the recently reported brexpipazole that was found to sensitize glioma stem cells to osimertinib via reducing survivin expression [3]. This type of generalized survivin inhibitors will not be reviewed in this article. Instead, based on the findings from the basic studies on survivin biology and molecular mechanism of action summarized in Tables 1 and 2, we will review the current cancer therapeutic strategy using survivin as a target, which can be classified into five categories: (i) Inhibitors that disrupt survivin interactions with its partner proteins; (ii) Inhibitors that disrupt survivin homodimerization; (iii) Inhibitors that decrease survivin gene transcription; (iv) Inhibitors that induce survivin mRNA degradation; and (v) Survivin or its peptide for immunotherapy. We will then summarize each of these survivin therapeutic strategies for facilitating future translational research related to drug discovery and cancer therapeutics by using survivin as a target.

### Inhibitors that disrupt survivin interactions with its partner proteins

#### Shepherdin and AICAR

**Fig. 1:**
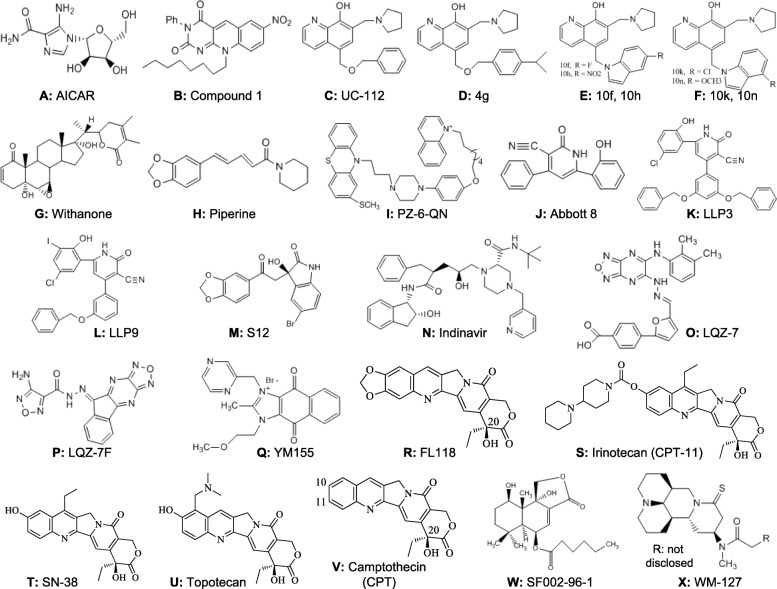
Chemical structure of various types of survivin inhibitors reviewed in this article is shown

The survivin inhibitors in this category must be based on a clearly defined molecular mechanism of survivin interaction with a partner protein. Shepherdin is the first example of this type of survivin inhibitors and was rationally designed in 2005 [4]. Shepherdin, a survivin ^79^KHSSGCAFL^87^ (minimum: ^79^KHSSG^83^) peptidomimetic agent, interrupts heat shock protein (Hsp) 90 interactions with survivin [4, 5]. Alternatively, shepherdin was incorporated into cancer cells using adenovirus-mediated expression systems, thus demonstrating a proof of principle for use of agents that disrupt survivin-Hsp90 binding as an anticancer agent [6, 7]. Since the peptide-mimetic approach has the inherent weaknesses of stability and delivery issues, a small molecule inhibitor, AICAR (Figure 1a), was identified and found to disrupt multiple Hsp90 client proteins including survivin [8]. These peptidomimetics occupy the Hsp90 ATP pocket to prevent survivin from binding to Hsp90. In this regard, shepherdin and AICAR may more appropriately be classified as Hsp90 inhibitors instead of as survivin inhibitors. In addition, AICAR is also known to be a weak AMPK and p53 activator [9, 10]. Nevertheless, although many promising Hsp90 inhibitors have been put on the shelf after extensive preclinical and clinical studies (e.g. 17-AAG, 17-DMAG, AUY922, KW-2478, STA-9090) in the past 20 years, recent studies demonstrated that Hsp90 inhibitors might still be useful for bolstering immunotherapy [11]. Therefore, further studies are needed and Hsp90-survivin inhibitor reposition may be possible, although the effect will likely not be specific to survivin.

#### Deazaflavin analog compound 1 and UC-112

The other examples in this category of survivin inhibitors are those that disrupt survivin interactions with Smac (also called DIABLO in mice) to induce apoptosis and cancer cell death. Several early studies indicated the importance of survivin-Smac interactions for cancer cell survival [12–16]. A screening of identifying small molecules based on an assay that disrupts survivin interactions with Smac or INCENP resulted in the finding of a small molecule of a 5-deazaflavin analog (Compound 1, Figure 1b) that can disrupt survivin-Smac interactions [17]. However, follow-up studies will be needed to identify the potential of Compound 1 antitumor efficacy if the authors intend to move Compound 1 and/or an analog into clinical trials.

By using the Smac N-terminus AVPI tetrapeptide as a template through a shape-based virtual screening against a drug-like compound library, a small molecule Smac mimetic named UC-112 (Figure 1c) was identified [18]. While it remains to be demonstrated as to whether UC-112 is able to disrupt survivin-Smac interactions, functional studies indicated that UC-112 is able to induce the activation of caspases 3, 7 and 9, and is able to selectively inhibit survivin protein in most of the cancer cell lines that were tested. The effect could be blocked in the presence of proteasome inhibitor MG-132 [18], suggesting that the UC-112 effects work through a proteasome degradation pathway. Follow-up studies identified a UC-112 analog 4g (Figure 1d) that exhibited 4-times better than UC-112 in the average IC50 (0.5 μM vs. 2.2 μM) tested in the NCI-60 panel cell lines. The 4g compound selectively degraded survivin at ≥1 μM in A375 and PC-3 cells, while XIAP, cIAP1, CIAP2 and Livin were largely unaffected [19]. Recently, this group reported the extensive chemical modification of UC-112 and found that the replacement of the benzyloxy moiety in UC-112 with an indole moiety was preferred to other moieties [20]. These authors identified additional 4 UC-112 analogs (10f, 10h, 10k, 10n, Fig. 1e, f) that maintained their unique selectivity against survivin among the IAP family members. *In vivo* study using 10f in a human A375 melanoma xenograft model revealed that 10f effectively inhibited melanoma tumor growth without observable acute toxicity [20]. These findings suggest that UC-112 is likely a good platform for deriving Smac mimetics survivin inhibitors. However, it would be intriguing to see whether the efficacy of these compounds is associated with the disruption of survivin-Smac interaction potential, which would strongly strengthen the mechanism of action for these compounds.

Additionally, computational molecular docking studies also identified other survivin inhibitors that were designed to disrupt survivin-Smac interactions. This includes withanone (Fig. 1g) [21] and piperine derivatives (Fig. 1h) [22]. However, experimental studies to verify the binding nature and property as well as the antitumor activity will be needed for a critical evaluation of these compounds.

Finally, just prior to submission of this article, a new small molecule PZ-6-QN (Fig. 1i) was identified, and shown to bind survivin similarly to the known Smac peptide, AVPI [23]. Cell-based mechanistic studies indicated that PZ-6-QN enters mitochondria to inhibit the survivin-Smac interaction and promotes the release of Smac and cytochrome c from mitochondria into cytosol; and importantly, PZ-6-QN exhibits good anticancer activity against various cancer cells including HeLa, A549, HCT116 and MCF-7 [23].

### Inhibitors that disrupt survivin homodimerization

#### Abbott 8, LLP3 and LLP9

Crystal structure analysis of both human and mouse survivin revealed that survivin forms a homodimer through a symmetric interaction of two survivin monomers along the molecular dyad axis [24–26], which is required for survivin protein stabilization for its function. This finding lays a foundation for designing compounds to disrupt survivin homodimerization for possible cancer therapeutics. The first set of compounds that bind to survivin at its dimerizing interface were identified in Abbott Laboratories, and a lead small molecule compound 8 (Abbott 8, Figure 1j) was identified [27]. Analogs were developed using computational modeling of the molecular interactions along the survivin dimerization interface [28]. This approach has led to promising survivin dimerization modulators. The two most potent survivin modulators, LLP3 and LLP9 (Figure 1k, l) caused major mitotic defects including delay of mitotic progression in proliferating human umbilical vein endothelial cells (HUVEC) and PC-3 prostate cancer cells at the concentration range of 50 nM to 100 nM [28]. However, although LLP3 was designed to bind to survivin at survivin homodimerization interface, *in vitro* binding studies indicated that LLP3 could disrupt the interaction of survivin with the small GTPase Ran, a critical regulator of bipolar mitotic spindle assembly, but not the survivin homodimer [29]. While this is consistent with the fact that LLP3 and LLP9 cause major mitotic defects [28], further studies will be needed to better understand the molecular mechanism of action. Additionally, a recent study indicated that LLP3 might be used in drug combination for treatment of colorectal cancer and the mechanism of action for LLP3 at least partially depends on XAF1 and p53 status [30]. Again, it is clear that further studies are needed to better understand the mechanism of action for Abbott 8 and LLP3-related compounds.

#### S12

A small molecule named S12 (Figure 1m) that targets the specific cavity adjacent to the survivin dimerization surfaces was identified through computational *in silico* screening, followed by chemical and biology studies [31]. S12 binding to survivin was confirmed by isothermal titration Calorimetry (ITC). Although it is unclear whether S12 disrupts survivin dimerization or disrupts a partner protein interaction with survivin monomer, S12 does alter spindle formation, cause mitotic arrest and cell death, and inhibit tumor growth [31].

#### Indinavir

Additionally, a virtual computational screen of database compounds was performed using a model built on the survivin dimerization/survivin-borealin interaction interface key residues; the authors identified the HIV protease inhibitor, indinavir (Figure 1n) as a potential compound that binds to survivin interface. However, indinavir may not actually bind to survivin, as the data shown in their report indicated that indinavir has no effect on survivin protein expression even at 510 μM for 48 hours, which is the IC50 of MDA-MB-231 breast cancer cell growth [32].

#### LQZ-7 and LQZ-7F

The most well documented survivin dimerization inhibitors were recently reported from Dr. Jian-Ting Zhang’s research group [33]. By using UCSF DOCK 6.0 Program [34], they performed an *in silico* dock screening of 200,000 compounds from the SPECS’s library (www.specs.net), to target the critical dimerization core residues Leu^98^ and Phe^101^ in the survivin dimeric interface [33]. One hundred compounds were selected on the basis of their GRID and AMBER score, Lipinski’s rule of five (drug likeness), and maximizing different clusters of compounds. Among the 100 selected compounds, 49 commercially available compounds were tested for cytotoxicity using Du145 and PC3 cells. They found that compounds 4, 7, 9, 12, 21, 36 and 42 are able to inhibit ≥50% survival in both cell lines at 20 μM. Importantly, compound 7 (named LQZ-7, Fig. 1o) exhibited a dose-dependent binding to survivin and disrupting survivin dimerization, while LQZ-7 had no effect on 14-3-3δ protein dimerization (control) [33]. These authors found that LQZ-7 accelerates proteasome-dependent degradation of survivin, without affecting survivin mRNA. However, LQZ-7’s IC50 in Du145 and PC3 cells is relatively high (~25 μM).

To improve LQZ-7 potency and reduce cytotoxicity, they searched the SPECS database and identified 6 commercially available LQZ-7 analogs (named LQZ-7A to F). Among these 6 new compounds, 5 have much lower IC50s than LQZ-7 [33]. Although LQZ-7B, LQZ-7C and LQZ-7F demonstrated similar abilities when it came to inducing survivin degradation, LQZ-7F (Fig. 1p) had a superior drug-like feature with a smaller molecule weight. Thus, the authors chose LQZ-7F for further in-depth studies and demonstrated that LQZ-7F binds to and degrades survivin through proteasome-dependent pathway [33]. Furthermore, they showed that LQZ-7F inhibits cell growth in a panel of cancer cell lines with IC50s of 0.4 – 4.4 μM, and induces 50-65% PC3 cell apoptosis at 5-10 μM in 24 hours [33]. LQZ-7F also disrupts microtubule structure and causes mitotic arrest [33]. Most importantly, these authors showed that by using 25mg/kg LQZ-7F via intraperitoneal injection once every 3 days for a total of eight treatments, LQZ-7F significantly inhibits PC3-established xenograft tumor growth by inhibiting survivin without inducing mouse body weight loss [33]. Again, this is the most convincing study among this category of survivin homodimerization inhibitors so far reported. However, the correlation of survivin expression level with LQZ-7F cytotoxicity among a panel of cancer cell lines was not very strong. This may have been due to the fact that different cancer cell types may require different amounts of survivin. Nevertheless, based on the current antitumor efficacy of LQZ-7F, the combination of LQZ-7F with other distinct mechanism-targeted drugs or other chemotherapeutic drugs warrants further studies. Alternatively, since LQZ-7F has the potential for further modification, LQZ-7F may serve as a platform for developing even better survivin homodimerization inhibitors.

One point that should be mentioned here is that survivin homodimerization for function is not always needed. A good example of this would be the survivin monomer interaction with the chromosomal passage protein, Borealin during mitosis. Borealin replaces one survivin monomer to become a survivin-Borealin heterodimer protein complex [35]. In fact, survivin homodimers prevent apoptosis, whereas the survivin monomer interaction with Borealin-INCENP during mitosis contributes to the control of cell division [36]. This may also be true for the survivin-ran interaction [29, 37]. However, this is not always the case, as most recent studies have indicated that during non-muscle myosin II (NMII)-mediated cytokinesis, only the survivin homodimer binds to NMII, attesting to the biological importance of survivin homodimerization [38]. Nevertheless, it would be intriguing to discover whether the currently identified survivin homodimerization inhibitors such as LQZ-7 and LQZ-7F could also disrupt survivin-Borealin or survivin-ran interactions. Theoretically speaking, it should, as Borealin occupies the survivin monomer dimerization site. This notion is also consistent with the finding that LQZ-7F also disrupts microtubule structure and causes mitotic arrest [33].

### Inhibitors that decrease survivin gene transcription

We have thoroughly reviewed the general term of agents that inhibit survivin expression [2]. We will now focus on reviewing the studies that used survivin promoter/regulatory sequence-driven reporter system to find survivin inhibitors through high throughput screening (HTS) of compound library. Thus, we could generally define the survivin inhibitors discovered in this way are inhibitors that downregulate the survivin gene transcription as the major mechanism.

#### YM155

YM155 (Fig. 1q) is the first small molecule that was discovered via HTS of in-house chemical compound libraries owned by Astellas Pharma (Japan) using the 2767bp survivin promoter (-2810 to -44, +1ATG translation site)-driven luciferase reporter system transfected in HeLa cells [39]. YM155 emerged as a small molecule that strongly inhibits survivin expression at both the protein and mRNA levels when used at 10-100 nM levels [39]. Inhibition of survivin by YM155 appeared to be highly selective, since YM155 did not inhibit the expression of cIAP2, XIAP, Bcl-2, Bcl-XL, Bad [39], or cIAP1, p53 and Stat3 [40] at the concentrations up to 100 nM. Another study revealed that YM155 strongly inhibited survivin promoter activity at ≥10 nM, while it showed only a minor inhibitory effect on the gene promoter activity of p21^cip1/waf1^, dihydrofolate reductase (DHFR), human thrombin receptor (HTR), and thymidine kinase (TK) at the same concentration [41]. Additionally, a systematic analysis of appropriate truncated survivin promoter-luciferase constructs plus DNA gel shift assay (EMSA) revealed that one mechanism by which YM155 inhibits survivin expression involves the abrogation of Sp1 function in the maintenance of constitutive survivin expression [41]. However, YM155 failed to decrease Sp1 protein expression or to interact with Sp1 DNA-binding elements. Instead, it was found that YM155 treatment is able to change Sp1 protein subcellular localization. This suggests that after YM155 treatment, the Sp1 protein is unable to access its DNA binding sites to maintain survivin transcription. Studies also revealed that the transcription factor, ILF/NF110, is a physiological target for YM155 [42]. YM155 treatment disrupted ILF3/p54nrb interaction and translocated ILF3 from the nucleoplasm to the nucleolus [43]. The interesting thing is that the subcellular re-localization of ILF3 is very similar to Sp1 re-localization after YM155 treatment [41, 43]. Therefore, there is a possibility that Sp1 and ILF3 may interact with each other to maintain survivin expression. In this scenario, YM155 treatment relocates the ILF3-Sp1 protein complex from the nucleoplasm to the nucleolus. This would result in both Sp1 and ILF3 being unable to access their DNA binding sites on the survivin promoter and thus diminishing survivin transcription. It will be intriguing to see the ultimate fate of YM155-targeted cells. For example, it remains unknown whether these YM155-targeted cells irreversibly enter apoptosis or whether any of them can survive after removal YM155. This question arises as there is no evidence to indicate that YM155 can decrease Sp1 or ILF3/p54nrb expression. It is unlikely that the Sp1 and ILF3/p54nrb stories reflect the entire mechanism of action for YM155.

Interestingly, later studies indicated that YM155 is able to inhibit Mcl-1 expression in PC-3 (prostate), H28 (mesothelioma), U251 and D37 (glioblastoma) cancer cells [44]. However, in pancreatic cancer cell lines, YM155 inhibits both survivin and XIAP without affecting the expression of Mcl-1 and Bcl-XL [45]. These findings suggest that the effect of YM155 on some of its targets in the IAP and Bcl-2 families could be cancer cell type-dependent. Interestingly, a study demonstrated that both YM155 and its structural analog NSC80467 induce a DNA damage response [46]; and a recent study even showed that YM155 inhibits topoisomerase 2α decatenation and topoisomerase 1 (Top1)-mediated cleavage of DNA, suggesting that YM155 inhibits the Top1 enzyme activity [47]. Nevertheless, YM155 was much better than NSC80467 in terms of its potential to inhibit the expression of survivin [46]. Together, it is likely that YM155 exerts its anticancer effects through multiple mechanisms.

A major concern for YM155 is its chemical stability. The studies in the initial report used YM155 via a 3-day continuous infusion per week for 2 weeks or via intravenous routes five times per week for 2 weeks; YM155 in such schedules significantly delay tumor growth with tumor regression as compared with control [39]. The reported experiment lasted for two weeks; it is unclear whether the tumor will be inhibited continuously without further YM155 treatment. It was shown that as soon as the 3-day infusion of YM155 stopped, YM155 in plasma and tumors rapidly decreased [39]. Nevertheless, these authors demonstrated that YM155 treatment decreases survivin expression in the tumor at both Day 3 and Day 7 time points tested when administrating via the 3-day continuous infusion at 10 mg/kg [39].

Additional preclinical studies indicated that YM155 was also shown to increase the sensitivity of human non-small cell lung cancer (NSCLC) to gamma-radiation. Combination of YM155 with gamma-radiation significantly delayed the growth of NSCLC tumor xenografts in nude mice than either treatment modality alone [40]. Similarly, using YM155 in combination with platinum compounds (CDDP or CBDCA) [48] or with docetaxel [49] via 3-day continuous infusion for 2 weeks or 7-day continuous infusion therapy significantly delayed the growth of NSCLC [48] and melanoma [49] xenograft tumors compared to either treatment modality alone. Additionally, YM155 reduced spontaneous metastases and significantly prolonged the survival of animals with metastatic tumors in an MDA-MB-231-Luc-D3H2-LN orthotopic model [50]. Similar results were obtained through combination of YM155 with rituximab in a human B-cell non-Hodgkin lymphoma [51] or through combination of YM155 with gemcitabine in human pancreatic cancer [52].

Although the outcome from YM155 pre-clinical studies appears to support moving YM155 into clinical trials as a single agent or in combination with other therapeutic agents as reviewed above, multiple Phase I and Phase II clinical trials demonstrated that YM155 exhibits very limited antitumor efficacy when used alone [53–58] or in combination with other cytotoxic therapeutic agents [59–62].

It is our view that the failure of YM155 in clinical trials could be due to the chemical instability of YM155; this instability in turn could have resulted in limited antitumor efficacy. Evidence of this was indicated in the pharmacokinetic (PK) studies. These studies showed that after the end of YM155 treatment of YM155, a rapid decrease of YM155 occurs in both serum and tumors [39]. Additionally, we do not know whether the YM155 inhibition of its targets was long lasting or whether the targets recovered as soon as YM155 was cleared from the body. For example, YM155 downregulated survivin through blocking Sp1 and ILF3/p54nrb-mediated constitutive expression of survivin [41–43]; this appears to remove the transcription factors (Sp1, ILF3/p54nrb) from the nucleoplasm to the nucleolus [43], instead of degradation of them; this could give cancer cells an opportunity to recover quickly after removal of YM155.

#### FL118

FL118 (Figure 1r) is another promising small molecule for cancer treatment that was discovered through HTS, followed by *in vitro* and *in vivo* hit-to-lead analyses [63].

Differing from the discovery of YM155 using the 2767bp (-2810 to -44, +1ATG) survivin promoter with co-transfection of a selection marker plasmid in HeLa cells [39], the 4080bp human survivin promoter from – 4079 to +1ATG translation site of survivin was cloned upstream of the luciferase reporter gene in a vector containing a pre-made neomycin gene cassette (selection marker). This manipulation resulted in a new vector of pNeoHScyc4.08-luc [64]. Cancer cells from colon (HCT116), lung (A549), breast (MCF7), prostate (PC-3) and ovary (2008) origins were then transfected with pNeoHScyc4.08-luc, and individual stable cancer cell clones were obtained via G418 selection [64]. Individual clones were then tested for luciferase modulation using the previously confirmed model ligands, hedamycin [65] and Hoechst 33342 [66] to validate individual cancer cell models. The validated cell models were expanded for both library preservation and compound library screening [64]. The advantage of these screening assay models are: (i) addition of the -43 to +1 regulatory sequence is important for finding versatile survivin inhibitors that can not only inhibit survivin transcription, but can also inhibit survivin cell cycle-regulation, mRNA stability and translation efficiency. This is because the -43 to +1 regulatory sequence has cell cycle regulatory DNA elements and is transcribed into survivin mRNA as 5’-untranslational sequence. These DNA elements are important for cell cycle-regulation of survivin expression [67], and the 5’-untranslational sequence in the survivin mRNA is important for the regulation of survivin mRNA stability and translation efficiency [64]. Additionally, the use of one vector (of note, DNA was linearized before transfection) including both reporter system and selection marker would result in more predictable cancer cell models after transfection and selection [64]. Finally, the use of multiple cancer cell type assay models [64] increases the chances of finding versatile survivin inhibitors by using them in series to avoid cell type-specific potential candidates of survivin inhibitor during the hit-to-lead selection process.

Initially, more than 3,000 structurally diverse compounds representing about 200,000 compounds were screened at a concentration of 1 μM in one cell model (HCT116-luc). About 250 hit compounds were further cross-tested at a series of concentrations from 0.001 to 1,000 nM in the other four cell models described above (A549-luc, MCF7-luc, PC-3-luc, 2008-luc). This resulted in 20 top-hit compounds, which showed inhibition of luciferase activity at a concentration range of 1 nM to 100 nM within 24 hours of treatment [63]. A total of 207 chemical structure analogs relevant to the 20 hit compounds were then analyzed for their inhibition of survivin promoter activity in the generated model cells in parallel with determination of cancer cell growth inhibition by each compound *in vitro* via MTT assay. These studies resulted in five compounds (FL113, FL118, FL155, FL174 and FL199) that showed strong inhibition of survivin promoter activity, survivin expression, and cancer cell growth. The five compounds were then tested using mouse models of human tumor (colon, head-&-neck). It turned out that while each of the five compounds show significant antitumor activity, FL118 was the top compound, possessing exceptional efficacy to eliminate human xenograft tumor without relapse over a period of 60 days in a high percentage of human tumors in animal models [63, 68]. Interestingly, 3-dimentional (3D) cell models were recently developed for testing FL118 and several of the FL118 analogs [69, 70]. However, whether the 3D cell models could replace the early stage *in vivo* animal testing for cost-effectiveness and selection of the future FL118 analogs remains to be investigated.

Several interesting features of FL118 are worth highlighting here. First, FL118 is structurally similar to irinotecan, SN-38 (active metabolite of irinotecan) and topotecan (Figure 1s, t, u). All of these compounds are camptothecin (CPT, Figure 1v) analogs. It is well known that the mechanism of action for camptothecin (CPT) compounds, including the two United States Food and Drug Administration (FDA)-approved drugs topotecan and irinotecan, use topoisomerase I (Top1) as their therapeutic target [71–75]. However, the concentration required for FL118 to show its Top1 inhibition activity is 100 to 1,000 fold higher than the concentration required for FL118 to inhibit both survivin promoter activity and cancer cell growth [63]. Furthermore, in contrast to the fact that CPTs show loss of antitumor activity when cancer cells reduced or lost Top1 expression/catalytic activity [71, 72, 76], the sensitivity of human xenograft tumors to FL118 is irrelevant to Top1 expression; FL118 shows high antitumor sensitivity and efficacy in human cancer with low/negative Top1 expression [77]. This is consistent with our findings that FL118 inhibition of cancer cell growth occurs at the high pM to low nM range; whereas its effects on Top1 activity require μM levels [63]. Therefore, while Top1 inhibition by FL118 may occur it is not the primary mechanism of action for FL118.

Second, while FL118 showed no inhibitory effects on the gene promoters of p21^cip1/waf1^, dihydrofolate reductase, human thrombin receptor, and thymidine kinase, FL118 selectively inhibits the expression of not only survivin, but also Mcl-1, XIAP and cIAP2 [63]. In contrast, SN-38 and topotecan exhibited 10-100 fold weaker to inhibit these proteins [63, 78]. Genetic silencing or overexpression of survivin, Mcl-1, XIAP and cIAP2 revealed their role in FL118 effectiveness [63, 68]. DNA microarray studies showed that FL118 does not inhibit the expression of cIAP1, Bcl-2, Bcl-XL, Bcl-2, Bcl2A1, Bcl-w, Bcl-B, Bcl2L12, Bcl2L13, Bcl-G and Bcl2L15 (unpublished data), indicating additional selectivity of FL118 in its molecular targets. Furthermore, FL118 also inhibits MdmX/Mdm4 [79], a critical oncogenic protein involved in p53 pathway, and ERCC6 [80], a critical regulator in DNA repair. Importantly, while FL118 downregulation of MdmX induced senescence in cancer cells with wild type p53, FL118 exhibits even higher efficacy to inhibit cell growth and induce apoptosis in cancer cells without functional p53 (mutated or null) [79]. Additionally, siRNA silencing of survivin showed no effects on the expression of Mcl-1, XIAP, and cIAP2 [63], suggesting that FL118 inhibition of survivin expression is independent of its role in the inhibition of Mcl-1, XIAP and cIAP2. The independent inhibition of multiple antiapoptotic gene products (survivin, Mcl-1, XIAP, cIAP2) is important as various combinations of these proteins are known to be simultaneously overexpressed in various stages of resistant cancers. While the entire mechanism will need further investigation, one strategy for FL118 to control the expression of multiple oncogenic proteins is that these gene promoters are controlled by a panel of transcription factors that highly overlap with those from the survivin promoter [81]. Recently, it was reported that in MDA-MB-231 breast cancer cells, FL118 suppressed the expression of vimentin while enhancing the expression of E-cadherin [82], suggesting that there was the potential for FL118 to inhibit epithelial-mesenchymal transition (EMT) and cancer cell invasion and metastasis. Together, these findings indicate that FL118 is likely a unique and versatile small molecule for various types of cancer treatment.

Third, irinotecan, SN-38 and topotecan are the substrates of efflux pump proteins ABCG2/BCRP [83–87] and Pgp/MDR1 [88–92]. In contrast, FL118 is not a substrate for them, and can bypass their resistance [78, 93]. Consistently, FL118 has a favorable pharmacokinetics (PK) profile (accumulated in tumor and rapidly cleared in blood stream) after intravenous administration [78] and is orally available [77]. It is the new trend of research to find anticancer agents that are not ABCG2 substrates instead of using ABCG2 inhibitor for combinational treatment [94] and FL118 possesses these features.

Fourth, FL118 both before and/or after formulation is highly stable and can be stored at room temperature or 4°C without issue. FL118 can be put in high temperatures such as at 50-80°C for the spray-dry process for at least a period of time without any issue. Finally, FL118 also overcomes a number of other common resistance factors such as cancer cells with mutated p53, mutated APC and/or overexpression of HdmX/MdmX [79] or Kras gene mutation (unpublished observation). Again, FL118 is orally available [77], accumulates in human tumors in animal model, and effectively overcomes irinotecan and topotecan-resistant human tumors in animal models [78].

Along with the versatile and unique features of FL118 summarized above, FL118 has shown striking antitumor activity in human tumor animal models [63, 68, 78, 80, 95, 96]. FL118 exhibited significantly superior antitumor activity when compared with FDA-approved anticancer drugs commonly used in clinical practice (irinotecan, topotecan, doxorubicin, 5-FU, gemcitabine, docetaxel, oxaliplatin, cytoxan and cisplatin) [63]. Additionally, FL118 is able to eliminate small and large human tumors without relapse in a high percentage of mice within the two-month experimental period [63, 68].

Recent studies indicate that FL118 targets cancer stem cells (CSCs) by inhibiting a number of CSC markers and drug resistant proteins in lung cancer [97]. FL118 preferentially targets and kills cisplatin-resistant pancreatic cancer cells, and inhibits spheroid formation of pancreatic cancer stem cells [80]. Studies from the *In vivo* animal models of human pancreatic cancer patient-derived xenograft (PDX) tumors indicated that alone, FL118 effectively eliminated PDX tumors, while FL118 in combination with gemcitabine (a first line pancreatic cancer drug) eliminated PDX tumors that showed resistance/non-sensitivity to FL118 and gemcitabine treatment [80]. Consistently, FL118 appears to use multiple mechanisms to induce pancreatic cancer killing as well [80, 98]. Furthermore, toxicity studies with FL118 at low, middle and high doses in beagle dogs indicated that at only the high dose, some of the 39 hematopoietic and biochemical parameters tested slightly changed without other FL118-related clinical observations including dog behavior, food consumption and body weights [80].

**Fig. 2:**
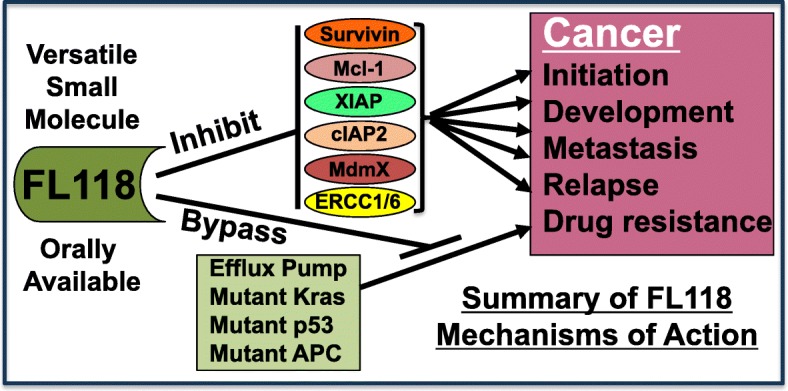
Summary of the experiment-supported FL118-relevant mechanisms of action: FL118 was demonstrated to (1) inhibit multiple cancer-associated survival and treatment-resistant proteins (survivin, Mcl-1, XIAP, cIAP2, MdmX, ERCC1/6); and (2) bypass additional treatment resistant factors (efflux pump proteins ABCG2 and Pgp, mutated Kras, mutated p53 and mutated APC)

In summary, FL118 has a number of attractive drug-like properties and is a versatile small molecule against cancer through multiple mechanisms of action (Fig. 2). Based on the current research progress and outcomes, FL118 will go into clinical trials with the indication of colorectal and pancreatic cancers in a year or so (personal communication).

#### SF002-96-1

Through use of the colorectal cancer cell line Colo320 transfected with the 1092bp (-1102 to -11, +1ATG) survivin promoter-driven luciferase report system to screen ~200 strains of imperfect fungi, a drimane sesquiterpene lactone (named SF002-96-1, Fig. 1w) was isolated in the fermentations of an Aspergillus species [99]. SF002-96-1 exhibited dose-dependent inhibition of survivin promoter-driven luciferase activity with a IC50 of 3.42 μM in parallel with the inhibition of survivin mRNA and protein expression, while showing no inhibitory effects on CMV or TOPFLASH promoter-driven luciferase activity [99]. Chromatin immunoprecipitation (ChIP) assay revealed that SF002-96-1 abrogates IL6-induced Stat3 activation or TNF-induced NF-κB activation-induced binding of their DNA sites in the survivin promoter in Colo320 cells. Finally, these authors demonstrated that SF002-96-1 inhibits Colo320 cell growth with an IC50 between 10.52 and 18,42 μM and induce apoptosis [99].

#### WM-127

The authors used a unique survivin transcription-controlled luciferase reporter system [100] when they discovered WM-127 (Fig. 1x). Specifically, the PCR-amplified 1097bp (-1097 to -1, +1ATG) survivin promoter was cloned into upstream of the EGFP cDNA reporter, and the PCR-amplified non-translated region of ~315bp from survivin exon IV immediately after the TGA stop codon was cloned into downstream of the EGFP cDNA reporter to make the survivin promoter/regulatory sequence-driven EGFP reporter system that at least partially mimic endogenous survivin gene regulation [100]. This vector was then cloned into lentiviral expression system and then using the lentiviral system-infected liver cancer HepG2 cells as a screening model containing Sur5P-EGFP-Sur3U reporter system for drug screening [100]. This is an improved survivin inhibitor selection system. Theoretically, this drug selection system would find survivin inhibitors that affect miRNAs or proteins that regulate survivin mRNA stability and translation by interacting with the survivin mRNA non-translated regions at the 5’- and/or 3’-ends. However, for making an ideal drug selection system, the system may include a ≥ 4kb survivin promoter, because initial studies [101] and also later investigation (unpublished observation) indicated that up to 4kb survivin promoter is important for the regulation of survivin expression. Additionally, the system may also include the entire 1.15kb survivin mRNA 3’-non-translated region, because this would find survivin inhibitors that affect miRNA and/or proteins that act on the entire 1.15kb survivin non-translated region for survivin mRNA stability and translation control. Nevertheless, by using this novel survivin inhibitor selection system, these authors screened over one hundred structurally modified matrine derivatives, the WM-127 was found to have the strongest ability to inhibit EGFP expression. Their studies indicated that WM-127 inhibits survivin protein and cell viability in a dose-dependent manner [100]. WM-127 has an IC50 about 52 μg/ml (of note, based on the WM-127 derivatives’ structure-calculated molecular weight, 52 μg/ml is about 122 μM) in HCC and induced HCC cell cycle arrest and apoptosis, and delayed HCC xenograft tumors in nude mice [100]. Mechanistic studies showed that WM-127 suppressed the activity of survivin/β-catenin pathway and induced the expression of Bax [100]. Overall, WM-127 is a prototype molecule with moderate anti-HCC tumor activity. Further improvement of WM-127 antitumor activity via chemical modification is required before moving forward for clinical trials.

Additionally, the human survivin 303bp core promoter (-300 to ATG translation site)-driven EGFP reporter vector was stably transfected into mouse embryonic stem cells D3 (ATCC CRL-1934) as a survivin expression reporter model for embryonic toxic drug screen [102]. Given the survivin promoter-relevant cancer cell models discussed above, this model requires considerable improvement before being reliably used for compound screening. Nevertheless, use of survivin promoter/regulatory sequence-driven reporter systems as an important strategy to discover versatile anticancer drugs that inhibit survivin as well as other important oncogenic targets, might gradually earn more attention and/or recognition in the coming years.

### Inhibitors that degrade survivin mRNA

Specific degradation of survivin mRNA for controlling survivin expression is a practical approach in modern technology and is important for cancer research as a useful tool for protein function analysis and also for potential cancer therapeutics.

For degradation of survivin mRNA, survivin antisense oligonucleotides (e.g. ISIS 23722) were used from the initial survivin studies that began two decades ago [103], followed by the use of ribozyme approach [104–106] and then the use of small interfering RNA (siRNA) [107–110]. However, after review of this specialized area, we found that while the ribozyme approach was used for some time in research [111–113], further studies toward the use of this approach for cancer therapeutics were not progressing; this is likely due to the much easier approach of siRNA technology being available. Consistent with this notion, the survivin siRNA approach was used throughout the history of survivin studies [114–118]; the survivin siRNA studies in recent years show a clear trend of developing and using various new delivery technologies of survivin siRNA with or without combination of a cancer drug for therapeutics *in vitro* [119–123] and *in vivo* [124–131]. Although clinical trials using a type of nanotechnology to deliver survivin siRNA with or without combination of a cancer therapeutic drug remain to be seen, survivin siRNA formulated in a novel delivery system as an anticancer product will likely come into clinical trials in the near future.

In contrast to the survivin ribozyme and siRNA approaches, there are two survivin antisense oligonucleotides, ISIS 23722/LY2181308 and SPC3042/EZN-3042 that have moved into clinical trials.

#### ISIS 23722/LY2181308

The survivin antisense DNA oligonucleotide ISIS 23722 (5’-**TGTG**CTATTCTGTG**AATT**-3’, the bolded bases are the 2**’**-O**-**methoxyethyl nucleosides) was initially identified as the most active oligonucleotides by screening of 40 2’-O-methoxyethyl chimeric phosphorothioate oligonucleotides for inhibition of survivin mRNA in T24 bladder carcinoma cells using real-time qRT-PCR [103]. ISIS 23722 was demonstrated to induce myeloid leukemia cell proliferation defects and cell death involving mitochondrial pathway [132].

ISIS 23722 was subsequently acquired by Eli Lilly and renamed LY2181308. LY2181308 was subsequently demonstrated to inhibit survivin expression, induce caspase-3 activation and apoptosis, and inhibit tumor growth *in vivo* [133]. The first-in-human PK studies of LY2181308 indicated that among 40 patients treated with LY2181308 at doses of 100 to 1000 mg, 26 patients were evaluated at the recommended Phase 2 dose (750 mg), and demonstrated that LY2181308 accumulated in tumor tissue, reduced survivin mRNA and protein expression by 20%, and restored apoptotic signaling in tumor cells *in vivo* and exhibited rapid tissue distribution and terminal half-life of 31 days [134]. A subsequent Phase I study in Japan indicated that when 14 patients with solid tumors unmanageable with standard therapy received LY2181308 at 400, 600 or 750 mg as a 3-h intravenous infusion for 3 consecutive days and thereafter once a week, the studies revealed common reversible grade 1/2 toxicities of a flu-like syndrome, prolonged prothrombin time, thrombocytopenia and fatigue [135]. The dose-limiting toxicity was reversible grade 3 elevation of ALT/AST/gamma-GTP in 1 patient treated at the 750-mg dose. PK analysis showed a terminal half-life of 21 days and an extensive tissue distribution of LY2181308 [135]. In 12 evaluable patients, one patient had stable disease, while the remaining 11 patients had progressive disease [135]. More clinical trials of LY2181308 also indicated a kidney injury risk [136] and that LY2181308 exhibited moderate tumor uptake with an up to 40% decreased tumor metabolism, and the highest uptake is in kidney and liver [137]. Although additional phase I clinical trials of LY2181308 alone or in combination did not exhibit exciting or promising results [138], two Phase II clinical trials of LY2181308 were still conducted in patients with NSCLC in combination with docetaxel [139] and in patients with castration-resistant prostate cancer (CRPC) in combination with docetaxel [140]. In the first Phase II study, comparison of the antitumor activity of LY2181308 plus docetaxel (n=108) with docetaxel alone (n=54) indicated that no improvement in antitumor activity between the two arms for progression-free survival (PFS) (2.83 months with LY2181308/docetaxel and 3.35 months with docetaxel) [139]. In the second Phase II study, patients with metastatic CRPC were randomly assigned to receive docetaxel (control arm, *n*=56) or the combination of LY2181308 with docetaxel (experimental arm, n=98). The study revealed that the median PFS of treated patients for the experimental arm was 8.64 months versus 9.00 months in the control arm. The median overall survival (OS) for the experimental arm was 27.04 months compared with 29.04 months in the control arm [140]. In the experimental arm, patients had a numerically higher incidence of grades 3-4 neutropenia, anemia, thrombocytopenia, and sensory neuropathy [140]. The outcomes from the two Phase II trials were very disappointing, leading to the discontinuation of clinical studies using LY2181308.

It is important to consider why the clinical trials of LY2181308 resulted in failure. While nobody can really know for certain, there are two reasons that may be considered. First, while the modified oligonucleotide increases oligonucleotide stability, the modification may result in higher toxicity *in vivo* due to its difficulty to be detoxified and eliminated from the body. If this is indeed one of the reasons, this is unlikely to be observed by using *in vitro* cell cultural studies (see below). Second, after the DNA oligonucleotide modification, its efficiency to induce survivin mRNA degradation may be compromised. This decrease of survivin mRNA degradation efficiency may be compensated or even hidden by the increased toxicity of the modified oligonucleotide itself to cancer cells. While the *in vitro* studies may not recognize these issues, head-to-head comparative clinical studies in cancer patients would make the hidden issue appear. Of course, there are other possibilities that may also exist.

#### SPC3042/EZN-3042

SPC3042 was initially developed by Santaris Pharma and is a 16-mer oligonucleotide (5’-**CTCA**ATCCATGG**CAG**C-3’) with a fully phosphorothiolated gapmer containing 7 locked nucleotides in the flanks (bolded). The first study showed that the stability of SPC3042 in mouse plasma is similar to LY2181308, but SPC3042 exhibited higher potency for survivin mRNA inhibition compared with LY2181308 [141]. Functional studies indicated that SPC3042-mediated downregulation of survivin leads to cell cycle arrest, pronounced cellular apoptosis, and a moderate downregulation of Bcl-2 [141]. It was also shown that SPC3042 is a sensitizer of prostate cancer cells to Taxol treatment *in vitro* and *in vivo* [141]. SPC3042 was subsequently acquired by Enzon Pharmaceuticals and renamed EZN-3042.

In a chemical-induced liver regeneration model, treatment with a mouse homolog of EZN-3042 resulted in 80% down-modulation of survivin mRNA [142]. In A549 and Calu-6 lung xenograft models, treatment with EZN-3042 single agent induced 60% inhibition of survivin mRNA in tumors and 37-45% tumor growth inhibition [142]. In Calu-6 model, when EZN-3042 was combined with paclitaxel, an 83% tumor growth inhibition was obtained [142]. It was also shown that knockdown of survivin using EZN-3042 in combination with chemotherapy eliminated drug-resistant acute lymphoblastic leukemia cells [143]. Subsequently, a Phase I study of EZN-3042 in pediatric patients with second or greater bone marrow relapses of B-lymphoblastic leukemia was conducted [144]. EZN-3042 was administered as a single agent on days 2 and 5, and then in combination with a 4-drug re-induction platform on days 8, 15, 22, and 29. At the dose level 1 (EZN-3042 2.5 mg/kg/dose, n=6), 1 patient developed a grade 3 of γ-glutamyl transferase elevation, and another patient developed a grade 3 of gastrointestinal bleeding [144]. Decreased survivin mRNA and protein expression was observed in 2 patients after EZN-3042 dosing from the assessed 5 patients [144]. The authors concluded that although some biological activity was observed, the combination of EZN-3042 with intensive re-induction of chemotherapy was not tolerated at a dose that led to consistent downregulation of survivin expression [144]. Therefore, the trial was terminated following the completion of dose level 1 and further clinical development of EZN-3042 was halted. However, in a canine lymphoma and osteosarcoma studies, it was shown that EZN-3042 inhibits growth, induces apoptosis and enhances chemosensitivity in canine lymphoma and osteosarcoma cells, and inhibits survivin transcription and protein production in orthotopic canine osteosarcoma xenografts [145]. These authors suggested that EZN-3042 might be further evaluated in dogs with cancer.

The failure of EZN-3042 appeared to be mainly due to toxicity. Whether the toxicity came from the unique locked modification of the EZN-3042 oligonucleotides, as discussed in the case of LY2181308, is unknown. Nevertheless, a breakthrough to find survivin mRNA inhibitors may come from the siRNA approach in combination with the development of nanotechnology for delivery [119–131], since survivin siRNA design in the coming years might take inspiration from various miRNA-mediated survivin mRNA inhibition. Currently miRNA regulation of survivin mRNA/transcripts is a hot research area, which has room for further in depth, extended studies (see Table 1) [146–149]. The current status of study in this area is that, while many miRNAs were found to bind to survivin mRNA/transcripts with defined inhibitory effects, many other miRNAs were found to bind to survivin transcripts/mRNAs without defined effects (Table 1), which calls for further investigation.

### Survivin or its peptides that are used for immunotherapy

Survivin-based cancer immunotherapy is also a research area of continuous interest; such research started closely following the initial survivin discovery and characterization [150–153] [for the details of the early work, see review [154]. The sustained interests of survivin immunotherapy originate from the observation that survivin is undetectable in all non-proliferative normal tissue and shows much lower level expression in proliferative normal tissues in comparison with the expression of survivin in cancer tissue [154]. Here, we update the cases that have been developed into survivin immunotherapy-related clinical trials. Studies on survivin epitope characterization in vitro will not be reviewed here.

#### Survivin-2B80-88

Following their initial finding [153], the survivin-2B80-88 (AYACNTSTL) vaccination-based phase I clinical study was conducted to assess patients with advanced or recurrent colorectal cancer that expresses survivin [155]. Vaccinations with survivin-2B80-88 were given subcutaneously six times to 15 patients at 14-day intervals; 3 suffered slight toxicities (anemia, grade 2; general malaise, grade 1; fever, grade 1). No severe adverse events (SAEs) were observed in these patients. Tumor marker levels (CEA and CA19-9) decreased transiently in 6 patients during the period of vaccination. Slight reduction of the tumor volume was observed in one patient. No changes were noted in three patients, while the other eleven patients experienced progressive disease (PD). Analysis of peripheral blood lymphocytes of one patient revealed an increase in peptide-specific cytotoxic T lymphocytes (CTLs) frequency from 0.09% to 0.35% of CD8+ T cells after 4 vaccinations. The authors conclude that survivin-2B80-88-based vaccination is safe and should be considered further for potential immune and clinical efficacy in HLA-A24-expression patients with colorectal cancer [155].

A Phase I clinical study of survivin-2B80-88 peptide vaccination in 9 patients with metastatic urothelial cancer (MUC) was subsequently conducted to further assess safety and efficacy [156]. A total of 46 vaccinations were carried out. There were no SAEs. HLA-A24/survivin-2B80-88 peptide tetramer analysis revealed a significant increase in the peptide-specific CTL frequency in five patients, after the vaccination was given. A slight reduction in tumor volume was observed in one patient [156]. The authors indicated that survivin-2B80-88 peptide-based vaccination is safe and should be considered further for potential immune and clinical efficacy in urothelial cancer patients as well [156]. This group then started another phase I clinical trial of survivin-2B80-88 vaccination in combination with interferon α (IFN-α) for MUC patients [157]. The studies indicated that a significant increase in the frequency of the peptide-specific CTLs was observed after vaccination, and of the enrolled 21 patients, 6 patients had stable disease and no SAEs were observed in any patients [157]. These authors summarized that the 30 MUO patients received survivin-2B80-88 vaccination in the 2 trials above had significantly better overall survival than a comparable control group of MUO patients without vaccination (P = 0.0009) and thus, survivin-2B80-88 vaccination may be a promising therapy for selected patients with MUC refractory to standard chemotherapy [157].

This group also ran a phase I clinical study to evaluate the safety and the efficacy of survivin-2B80-88 peptide vaccination in HLA-A24-positive patients with advanced or recurrent oral cancer [158]. From 11 enrolled patients, 10 patients that completed the vaccination protocol reported no adverse events (AEs). In two patients, the levels of serum squamous cell carcinoma (SCC) antigen decreased transiently during vaccination. Tumor regression compatible with a partial response (PR) was noted in one patient and the remaining nine patients experienced PD [158]. An increase of the peptide-specific CTL frequency was immunologically detected in six of the eight patients. These authors concluded that survivin-2B80-88 peptide vaccination was safe and had therapeutic potential for oral cancer patients [158].

Since the use of survivin-2B80-88 peptide alone for immunotherapy was unable to elicit enough effects for routine clinical use, these authors assessed survivin-2B80-88 plus incomplete Freund's adjuvant (IFA) versus survivin-2B80-88 plus IFA and IFN-α for clinical trials in patients with advanced colon cancer [159]. The trial indicated that although the effect of survivin-2B80-88 plus IFA was not significantly different from that with survivin-2B80-88 alone, treatment with survivin-2B80-88 plus IFA and IFN-α resulted in clinical improvement and enhanced immunological responses from patients, and survivin-2B80-88 peptide-specific CTLs increased at least twofold in four of eight patients [159]. Subsequent studies of single-cell clone separation by cell sorting of peptide-specific CTLs showed that each CTL clone was not only peptide-specific but also cytotoxic against human cancer cells in the context of the expression of both HLA-A24 and survivin molecules [159]. Based on these observations, this group then performed another Phase I clinical trial of survivin-2B80-88 plus IFA and IFN-α in patients with advanced pancreatic cancer. The studies indicated that more than 50% of the patients had positive clinical and immunological responses, while no obvious increase of the frequency of survivin-2B80-88-specific CTLs were observed in patients administrated only IFN-α [160].

Based on these promising results, a multicenter randomized phase II study in patients with advanced pancreatic adenocarcinoma was conducted [161]. Specifically, patients after gemcitabine and/or tegafur-gimeracil-oteracil (TS-1) were randomly assigned into 3 groups and treated with (i) survivin-2B80-88 plus IFNβ (n=30), (ii) survivin-2B80-88 only (n=34), or (iii) placebo (*n*=19) until the patients showed disease progression. The studies revealed that no significant improvement in PFS was observed for the patients who were vaccinated with survivin-2B80-88 plus IFN-β. However, survivin-2B80-88-specific CTLs were significantly increased in the survivin-2B80-88 plus IFN-β group. Additionally, some patients participated in a subsequent round of 4 treatments with survivin-2B80-88 plus IFN-β (Step 2). Those patients who had received survivin-2B80-88 plus IFN-β in Step 1 and Step 2 showed better overall survival (OS) compared with those who had received placebo in Step 1 [161]. Based on this result, a longer survivin-2B80-88 plus IFN-β vaccination protocol might confer survival benefit.

In summary, in comparison with the promising Phase I studies with survivin-2B80-88 plus IFN-α vaccination reviewed above, the Phase II outcomes somewhat indicated that additional Phase II studies would be needed for a clear conclusion. However, it is unclear why in the Phase II clinical trial, survivin-2B80-88 plus IFN-β replaced survivin-2B80-88 plus IFN-α used in Phase I clinical trials. Whether this may play a role for outcomes is unclear.

#### SurVaxM

SVN53-67 contains multiple HLA-A*02 epitopes and antigen-binding motifs for various HLA-A or HLA-B [162]. Preclinical studies identified a KLH-conjugated survivin peptide mimic SVN53-67/M57-KLH (SurVaxM) that stimulates immune response against murine glioma in vivo and human glioma cells in vitro was used to test safety, immunogenicity and clinical effects in glioma patients in a clinical study [162]. Recurrent malignant glioma patients with survivin-positive tumors and having either HLA-A*02 or HLA-A*03 MHC class I allele-positivity were given subcutaneous injections of SurVaxM (500 μg) in Montanide ISA 51 with sargramostim (100 μg) at 2-week intervals [162]. The trial indicated that SurVaxM is well tolerated with mostly grade 1 AEs and no SAEs attributable to the study drug. Six patients experienced local injection site reactions; three patients reported fatigue (grades 1 and 2), and two patients experienced myalgia (grade 1). Six of eight immunologically evaluable patients developed both cellular and humoral immune responses to vaccine. The vaccine also stimulated HLA-A*02, HLA-A*03 and HLA-A*24-restricted T cell responses. Three patients maintained a PR or stable disease (SD) for more than 6 months. Median PFS was 17.6 weeks, and median OS was 86.6 weeks from study entry with seven of nine patients surviving more than 12 months [162].

In the American Society of Clinical Oncology (ASCO) Annual Meeting (June 2019), results were presented from a five center single-arm Phase II clinical trial of the immunotherapy vaccine SurVaxM in combination with standard therapy (craniotomy, radiation, and treatment with temozolomide both before and after surgery) that was conducted in patients with newly diagnosed glioblastoma (nGBM) [163]. Specifically, 63 nGBM patients (ages 20-82, median 60) underwent craniotomies with near-total resection (<1cm^3^ residual contrast enhancement), temozolomide chemoradiation, adjuvant temozolomide and SurVaxM. Patients received 4 doses of SurVaxM (500 μg) in Montanide with sargramostim (100 μg) biweekly, followed by maintenance SurVaxM with adjuvants every 12 weeks until tumor progression. The median time to first immunization was 3.0 months (range 1.9-4.0) from diagnosis. Survivin expression in nGBM tumor ranged from 1-40% (median 12%) by immunohistochemistry. The studies revealed that there is no regimen limiting toxicity or grade ≥ 3 SAE attributable to SurVaxM. The most common AE was grade 1-2 injection site reactions. 12-month OS (OS12) was 86% from first immunization and 93.4% from diagnosis. OS12 for methylated O (6)-methylguanine-DNA-methyltransferase (MGMT, associated with temozolomide sensitivity) was 93.1% and un-methylated MGMT (associated with temozolomide resistance) was 78% from first immunization. The median time to tumor progression (i.e. mPFS) was 13.9 months from diagnosis. The median OS remains to be reached. SurVaxM produced an increase in survivin-specific IgG titer from pre-vaccine baseline to ≥ 1:10,000 in 67% of patients and ≥ 1:100,000 in 27%. CD8+ T cell responses were observed. Anti-survivin IgG and OS were correlated [163]. Based on the results, these authors concluded that SurVaxM immunotherapy generated encouraging efficacy and immunogenicity in nGBM and has minimal toxicity [163].

Here, we should mention that survivin peptide immunogen-reactive antibodies should be considered as an additional advantage for survivin immunotherapy. The potential of this concept has been recently demonstrated in a couple of studies explored from different angles [164, 165].

#### Other survivin peptides in cocktail

Based on the findings from the initial studies [151, 152], vaccination of 3 modified cocktail peptides [FTELTLGEF (HLA-A1), LMLGEFLKL (HLA-A2) and EPDLAQCFY (HLA-B35) using 3 vaccination regimens against survivin in 61 HLA-A1/-A2/-B35-positive patients with treatment-refractory stage-IV metastatic melanoma were conducted in a Phase II trial; 55 patients were evaluable for response and survival, and 41/55 for survivin-specific T-cell reactivity (SSTR). Patients achieving progression arrest [complete response (CR) + PR + SD] more often showed SSTRs than patients with disease progression (p = 0.0008). Patients presenting SSTRs revealed a prolonged OS (median 19.6 vs. 8.6 months; p = 0.0077); multivariate analysis demonstrated SSTR as an independent predictor of survival (p = 0.013). The induction of SSTRs was associated with gender (female vs. male; p = 0.014) and disease stage (M1a/b vs. M1c; p = 0.010), but not with patient age, HLA type, performance status, or vaccination regimen [166]. These authors concluded that survivin-specific T-cell reactivity strongly correlates with tumor response and patient survival [166], indicating that vaccination with survivin-derived peptides is a promising treatment strategy.

EMD640744 is a 5-peptide cocktail of equal weight from different regions of the survivin protein polypeptide, which bind HLA-A1, A2, A3, A24, or B7. This survivin peptide cocktail in Montanide ISA 51 VG promotes anti-survivin T-cell responses in patients with solid cancers [167]. Among the 49 patients who received ≥2 EMD640744 injections with available baseline and ≥1 post vaccination samples, 31 patients (63%) obtained vaccine-activated peptide-specific T-cell responses. No dose-dependent effects were observed. In the majority of patients (61%), anti-survivin responses were detected only after vaccination, providing evidence for de novo induction. The best overall tumor response was SD (28%). EMD640744 was well tolerated; local injection-site reactions constituted the most frequent AE [167]. The authors concluded that vaccination with EMD640744 elicited T-cell responses against survivin peptides in the majority of patients [167], demonstrating the immunologic efficacy of EMD640744.

Using the previously reported survivin peptide cocktail [166, 167], the authors formulated the survivin peptide cocktail in a novel and strongly immunogenic vaccine platform called DepoVax^TM^ to form DPX-Survivac [168]. A Phase I clinical trial to test the safety and immune potency of DPX-Survivac in combination with immune-modulator metronomic cyclophosphamide in ovarian cancer patients was conducted [168]. All of the patients receiving the therapy produced antigen-specific immune responses; higher dose vaccine and cyclophosphamide treatment generated significantly higher magnitude responses [168]. Strong T cell responses were associated with differentiation of naive T cells into central/effector memory (CM/EM) and late differentiated (LD) polyfunctional antigen-specific CD4+ and CD8+ T cells [168]. Based on the Phase I outcome, the authors indicated that this approach enabled rapid de novo activation/expansion of vaccine antigen-specific CD8+ T cells and provided a strong rationale for further testing to determine clinical benefits associated with this immune activation, and that their data represent vaccine-induced T cell activation in a clinical setting to a self-tumor antigen [168].

Using the previously identified survivin peptide epitope Sur1M2 (LMLGEFLKL) [151, 152] and an indoleamine 2,3-dioxygenase (IDO) peptide (ALLEIASCL) as epitopes for vaccination in combination of the chemotherapy temozolomide, a Phase II study was conducted in metastatic melanoma patients [169]. Specifically, HLA-A2 positive patients with advanced malignant melanoma were treated biweekly with 150 mg/m^2^ temozolomide daily for 7 days followed by subcutaneous vaccination with 250μg/250μg of Sur1M2/IDO peptides in 500 μL Montanide solution at day 8. GM-CSF cytokine was used as an adjuvant, and 5% topical imiquimod cream was applied prior to vaccination. Treatment was repeated biweekly for a period of up to 6 months. Patients still benefiting from treatment would continue temozolomide biweekly and vaccination injections every 4 weeks until disease progression. The studies indicated that a total of 17 patients treated resulted in a clinical benefit rate of 18% including one patient with partial tumor regression [169]. Immune analyses revealed a vaccine specific response in 8 (67%) of 12 patients tested, a significant decrease in the frequency of CD4+ T-cells during treatment, a tendency towards decreasing frequencies of naive CD4+ and CD8+ T-cells, and increasing frequencies of memory CD4+ and CD8+ T-cells. Based on these results, the authors concluded that vaccine-induced immunity towards survivin and IDO-derived peptides can be achieved in combination with temozolomide in patients mainly suffering from grade M1c melanoma including patients with brain metastases. However, at the same time, these authors also indicated that significant clinical activity could not be proven in this small cohort study and a larger setup is needed for a more proper assessment [169].

Additionally, a study of an HLA-DR restricted survivin-derived CD4+ T cell epitope in a multi-peptide cocktail immunotherapy trial for prostate carcinoma patients indicated that the survivin peptides are promiscuously presented by several human HLA-DRB1 molecules, and they are naturally processed through dendritic cells. In vaccinated patients, it was able to induce frequent, robust and multifunctional CD4+ T cell responses [170]

Finally, based on the previous finding of the survivin or survivin peptide-derived HLA class I-restricted CD8+ T-cell epitopes [150, 152, 171, 172], 3 long survivin peptides, 17-34 (18aa), 84-110 (27aa) and 122-142 (21as) that cover all of the previously identified epitopes were used as a cocktail for vaccination [173]. Studies in healthy individuals showed that CD4+ and CD8+ T-cell immunogenicity of the survivin peptide cocktail happened in humans, irrespective of the individual's HLA types. High frequencies of spontaneous T-cell precursors specific to the survivin peptide cocktail were also detected in the blood of various cancer patients [173], demonstrating the absence of tolerance against these peptides. These authors further showed that the survivin peptide cocktail vaccine has high therapeutic efficacy against four different murine tumor models established, and is associated with vaccine capacity to generate both specific cytotoxic CD8+ and multifunctional Th1 CD4+ T-cell responses [173]. When tumors were eradicated, generated memory T-cell responses protected against re-challenge, allowing long-term protection against relapses [173]. Treatment with the survivin peptide cocktail vaccine was also found to reshape the tumor microenvironment by increasing the tumor infiltration of both CD4+ and CD8+ T cells but not Treg cells, therefore tipping the balance toward a highly efficient immune response [173]. These authors highlighted that this survivin long peptide cocktail-based survivin vaccine appears to be a promising cancer vaccine strategy and warrants further clinical development [173].

## Is there a strategy that leads to a breakthrough in survivin therapeutics?

The expression pattern and the multiple important functions of survivin through diverse mechanisms of action [1] (Tables 1 and 2) supports the targeting of survivin for cancer therapy. As reviewed above, we may find that each of the five survivin-therapeutic strategies has its advantages and disadvantages. We would like to discuss the 5 strategies below. Our ideas and/or opinions on these topics may contain bias and may be incorrect, but we hope that this would encourage a broad discussion of relevant topics in the field to form a revised mindset for the benefit of future translational research that is aimed at finding superior survivin-relevant antitumor agents for cancer therapeutics.

In order to discover survivin-partner protein interaction-disruptive inhibitors and survivin homodimerization-disruptive inhibitors, we have many modern technologies to create computational docking models for *in silico* selection of such inhibitors. This would allow us to find good drugs economically. The inhibitors discovered in this way could be highly specific with few off-target effects. Such inhibitors have a better possibility of becoming useful research tools that can be used to enrich our knowledge of survivin biology, even if we eventually discover that the inhibitors do not possess sufficient antitumor efficacy for cancer mono-therapy. Nevertheless, the potential low toxicity features of such survivin inhibitors have a great potential for combination treatment with other therapeutic drugs that have distinct or overlapped mechanisms of action. An important question that we asked ourselves is whether we would be able to find small molecules that have both high antitumor efficacy and high specificity to disrupt survivin homodimerization or interactions with other partner proteins. It remains to be seen whether this can be achieved. We acknowledge that some degree of luck always plays a role in achieving such success. However, the success of past efforts on Bcl-2 inhibitors encourages further studies. This includes the discovery of Bcl-2 inhibitors ABT-737 in 2005, Obatoclax in 2007, Navitoclax in 2008 and Venetoclax (ABT-199/GDC-0199) in 2013. Importantly, such drugs can be moved relatively quickly into the clinic, once discovered. For example, Venetoclax was approved by the FDA for treatment of chronic lymphocytic leukemia (CLL) in 2016 [174].

When it comes to the task of finding survivin gene transcription inhibitors, it is our view that it would be nearly impossible to find a small molecule that exclusively inhibits survivin transcription, although we do know now that selectively inhibiting survivin is possible. As reviewed earlier, by the use of survivin promoter/regulatory sequence-driven reporter system for HTS of compound libraries, it is highly possible to find small molecules that selectively inhibit not only survivin transcription, but also additional important cancer-associated proteins that share similar transcription control mechanisms. In this regard, YM155 and FL118 are the typical examples; both of them inhibit survivin as well as other oncogenic proteins. Of course, whether a promising candidate can successfully become a drug for cancer treatment would depend on many factors. This includes, but is not limited to, drug stability, PK profiles, *in vivo* availability, toxicity profiles and so on. It is our view that two important factors would affect the success of finding high efficacy inhibitors through this approach. One factor would be the use of the right survivin promoter/regulatory sequence to generate the reporter system to be transfected into multiple cancer cell types for compound selections. The other factor would be the use of the right compound libraries, which have the right structural diversity and a sufficient number of compounds for HTS. If these factors are present, then the chance of finding good hits will significantly increase. Of course, the type of reporter used would be another factor; both luciferase reporter and EGFP reporters were used. While EGFP could provide a convenient method for detection, luciferase could provide much more sensitivity and a wider dynamic range for compound identification. For these reasons, we prefer to use a luciferase reporter instead of EGFP for the HTS processes. Finally, another advantage of using the survivin promoter/regulatory sequence-driven reporter system to discover anticancer agents is that this strategy provides the possibility of finding unique and versatile small molecule inhibitors. These small molecule inhibitors may not only inhibit survivin transcription but also interact and inhibit protein regulators that control survivin mRNA stability and even translation processes, given that appropriate survivin promoter/regulatory sequence-driven reporter systems are used. Again, luck always plays some role in drug discovery and development.

When it comes to finding survivin mRNA inhibitors, the use of survivin DNA oligonucleotide to specifically degrade survivin mRNA has not led to good outcomes in clinic trials, primarily due to low efficacy and/or high toxicity. These disappointing results may be due to many different factors. As discussed earlier, one possibility may arise from the special modification of the oligonucleotides, which causes a decrease in efficacy and increase in toxicity in clinical trials (due to its difficulty to be cleared from the body, for example). The failure may also be resulted in part from the only partial inhibition of survivin mRNA by survivin antisense oligonucleotide, which may exhibit insufficient efficacy. Nevertheless, based on the siRNA reviewed earlier and the study status of miRNA presented in Table 1, we believe that nanotechnology delivery-mediated siRNA therapeutics may give us hope for this type of survivin inhibitory drugs to be developed in the coming decade. As an encouraging example, the US FDA and European Commission (EC) recently approved a siRNA drug, ONPATTRO (Patisiran) developed in Alnylam Pharmaceuticals for the treatment of patients with the polyneuropathy of hereditary amyloid transthyretin (hATTR)-mediated amyloidosis [175]. It is likely that the survivin siRNA and miRNA studies will have further developments in the coming years. It is possible that we could obtain inspiration from the miRNA regulation of survivin mRNA studies. Thus, we can design versatile siRNA that could selectively degrade not only survivin mRNA, but also degrade other oncogenic protein mRNA. This is likely the case for most (if not all) miRNAs that were found to inhibit survivin mRNAs/transcripts (Table 1), as reviewed earlier.

We reviewed the major findings of survivin immunotherapy in detail by focusing on those that have been moved into clinical trials. Survivin immunotherapy will continue to be a busy area of research in the coming years. This is mainly due to its potential nontoxic nature, as well as its unique cancer treatment approach of stimulating the immune system and inducing CTL production to kill cancer cells. Based on the current outcome, while survivin immunotherapy alone may not be sufficient to effectively manage cancer, this approach provides a great opportunity for combination treatment not only with standard therapies, but also potentially with targeted precision medicine as well.

## Conclusions

Survivin remains a promising target and biomarker for drug discovery and cancer therapeutics. The approach to discovering inhibitors that disrupt survivin-partner protein interactions or disrupt survivin homodimerization would derive small molecules that specifically disrupt survivin protein-protein interactions and thus, inhibiting survivin function and/or inducing survivin protein degradation. Similarly, survivin mRNA inhibitors, such as survivin antisense oligonucleotide or siRNA could specifically degrade survivin mRNA. Such survivin-specific inhibitors are expected to have low toxicity *in vivo* models and in human. Nevertheless, while it remains to be seen whether the inhibitors that specifically disrupt survivin protein-protein interactions could exhibit sufficient antitumor efficacy when used alone, the success of the Bcl-2 inhibitor [174] and siRNA [175] drugs for human disease treatment gives us encouragement, despite the fact that clinical trials of survivin antisense oligonucleotides obtained disappointing results, which may stem from irrelevant reason(s) as discussed early. In the case of survivin immunotherapy, it has been demonstrated that survivin peptide-mediated immunotherapy exhibited low toxicity in clinical trials and can increase survivin peptide-specific CTLs for patients to kill cancer cells. Based on the outcomes from the updated studies, it appears that survivin immunotherapy alone might be insufficient for effective cancer management. However, there is a great potential for survivin immunotherapy in combination either with standard therapy or possibly with targeted precision medicine. In contrast, since survivin is a great target and biomarker, the use of cancer cell-based survivin promoter/regulatory sequence-driven reporter system has the potential to provide a better possibility to find unique and versatile small molecules beyond only inhibition of survivin and thus, such small molecules may exhibit high anticancer efficacy with low toxicity to normal tissue due to cancer-associated or focused inhibition. Additionally, if the right reporter system and right compound libraries are used in the initial HTS step, this approach has the potential to find small molecule inhibitors that not only downregulate survivin transcription but also affect survivin mRNA stability and translation process through inhibiting the protein regulators that control survivin mRNA stability and/or translation processes.

## Data Availability

The summarized information presented in Tables 1 and 2 was retrieved from the GeneGo database at https://portal.genego.com/ (This is not a free website, access need to make a payment or institutional licensing) and organized by the authors. Relevant references related to the summarized information can be found in the database from the corresponding molecules under the “link info”.

## Abbreviations

AE: Adverse event
BIRC5: Baculoviral IAP repeat-containing protein 5
CPT: Camptothecin
CR: Complete response
CRPC: Castration-resistant prostate cancer
CTL: Cytotoxic T lymphocyte
FDA: Food and Drug Administration
Hsp: Heat shock protein
HTS: High throughput screening
IAP: Inhibitor of apoptosis protein
IFA: Incomplete Freund’s adjuvant
IFN: Interferon
miRNA: micro-RNA
MUC: Metastatic urothelial cancer
NSCLC: Non-small cell lung cancer
OS: Overall survival
PFS: Progression-free survival
PK: Pharmacokinetics
PR: Partial response
SAE: Severe adverse event
SCC: Squamous cell carcinoma
SD: Stable disease
siRNA: Small interfering RNA
SVN: Survivin
